# Investigating BB0405 as a novel *Borrelia afzelii* vaccination candidate in Lyme borreliosis

**DOI:** 10.1038/s41598-021-84130-y

**Published:** 2021-02-26

**Authors:** M. J. Klouwens, J. J. Trentelman, J. I. Ersoz, F. Nieves Marques Porto, R. Sima, O. Hajdusek, M. Thakur, U. Pal, J. W. Hovius

**Affiliations:** 1 Department of Internal Medicine, Center for Experimental and Molecular Medicine, Academic Medical Center, University of Amsterdam, Meibergdreef 9, 1105 AZ Amsterdam, The Netherlands; 2 Division of Infectious Diseases, Department of Internal Medicine, Academic Medical Center, Amsterdam, The Netherlands; 3 Amsterdam Multidisciplinary Lyme Borreliosis Center, Academic Medical Center, Amsterdam, The Netherlands; 4 Biology Centre, Institute of Parasitology, Czech Academy of Sciences, Ceske Budejovice, Czech Republic; 5 Department of Veterinary Medicine, University of Maryland, College Park and Virginia- Maryland Regional College of Veterinary Medicine, College Park, MD USA

**Keywords:** Immunology, Disease prevention

## Abstract

BB0405 is a surface exposed *Borrelia burgdorferi* protein and its vaccination protected mice against *B. burgdorferi* infection. As BB0405 is highly conserved across different *B. burgdorferi* sensu lato species, we investigated whether vaccination with recombinant BB0405 or through intradermal *bb0405* DNA tattoo vaccination could provide protection against different *Borrelia* species, specifically against *Borrelia afzelii*, the predominant *B. burgdorferi* sensu lato genospecies causing Lyme borreliosis across Eurasia. We immunized C3H/HeN mice with recombinant BB0405 or with a codon-optimized *bb0405* DNA vaccine using the pVAC plasmid and immunized corresponding control groups mice with only adjuvant or empty vectors. We subsequently subjected these immunized mice to a tick challenge with *B. afzelii* CB43-infected *Ixodes ricinus* nymphs. Upon vaccination, recombinant BB0405 induced a high total IgG response, but *bb0405* DNA vaccination did not elicit antibody responses. Both vaccine formulations did not provide protection against *Borrelia afzelii* strain CB43 after tick challenge. In an attempt to understand the lack of protection of the recombinant vaccine, we determined expression of BB0405 and showed that *B. afzelii* CB43 spirochetes significantly and drastically downregulate the expression of BB0405 protein at 37 °C compared to 33 °C, where as in *B. burgdorferi* B31 spirochetes expression levels remain unaltered. Vaccination with recombinant BB0405 was previously shown to protect against *B. burgdorferi* sensu stricto. Here we show that vaccination with either recombinant BB0405 (or non-immunogenic *bb0405* DNA), despite being highly conserved among *B. burgdorferi* sl genospecies, does not provide cross-protection against *B. afzelii*, mostly likely due to downregulation of this protein in *B. afzelii* in the mammalian host.

## Introduction

Lyme borreliosis is the most common vector-borne disease in the Northern hemisphere and is caused by spirochetes belonging to the *Borrelia burgdorferi* sensu lato (sl) group. They are transmitted by *Ixodes* ticks and although humans get infected by *B. burgdorferi* sl*,* they are accidental hosts and do not play a role in the spirochete’s enzootic life cycle^1^. Because *B. burgdorferi* sl are extracellular pathogens, the outer membrane of the spirochete, containing multiple surface exposed lipoproteins, is continuously exposed to the immune system of the host^2,3^. Many studies have focused on identifying new *B. burgdorferi* outer surface proteins (Osps), because these are key targets for the host’s humoral immune response and thus may be potential new vaccinogens^4^. Indeed, multiple protective Osps have been identified and OspA formed the basis of the only anti-Lyme vaccine that was publically available^5^. There is however a wide genetic diversity among *B. burgdorferi* sl genospecies and the spirochetes change surface proteins throughout their life cycle, which makes it challenging to identify protective antigens^1^.

Originally, Brooks et al. identified several surface-exposed *B. burgdorferi* sensu stricto (ss) outer membrane proteins to which specific anti-*B. burgdorferi* antibodies were shown to be bactericidal^6^. Among these proteins was BB0405, an outer membrane protein unique for *B. burgdorferi* sl species. With 78 to 90% identity between BB0405 orthologues, its sequence is highly conserved among *B. burgdorferi ss, B afzelii and B garinii*, the three major genospecies causing Lyme borreliosis^7^. Although initially thought not to be immunogenic during natural infection^8^ it was shown by Brooks et al. that BB0405 is immunogenic and actively expressed during nonhuman primate infection by *B. burgdorferi* by detecting the protein with sera from infected baboons^6,8^*.* Multiple studies show that BB0405 is necessary for establishing infection in mice, since *bb0405*-deletion mutants are unable to be transmitted from ticks and establish infection in mammalian hosts^7,8^. Of importance, vaccination with recombinant BB0405 also protected mice from *B. burgdorferi* infection by *B. burgdorferi*-infected ticks^7,8^. Thus, *bb0405* is a highly conserved antigen with the potential to form the basis for a vaccine protecting against multiple *B. burgdorferi* sl genospecies.

Most research on new Lyme vaccines focuses on recombinant proteins, but DNA vaccination constitutes an alternative vaccination platform^9^. For instance, a previous study by Wagemakers et al. has shown that DNA vaccination by tattoo with *B. afzelii* strain PKo Outer surface protein C (OspC) was fully protective against *B. afzelii* challenge in mice and induced favorable humoral immune responses compared to recombinant protein vaccination^10^. In line with this, we were able to show protection against *B. burgdorferi* strain N40 in a similar set-up, in which OspC from *B. burgdorferi* strain N40 was used both as recombinant as well as DNA vaccine (Klouwens et al. manuscript in preparation).

In the current study we aimed to investigate the role of BB0405 in providing protection across *B. burgdorferi sl* genospecies. To this end, mice were immunized with *B. burgdorferi B31*-derived recombinant BB0405 or *bb0405* DNA vaccine and subsequently challenged with *B. afzelii* CB43-infected ticks, after which immunogenicity and host protection of the two different vaccination approaches were determined using established methods.

## Results

### Immunogenicity of *bb0405* antigens

**Figure 1:**
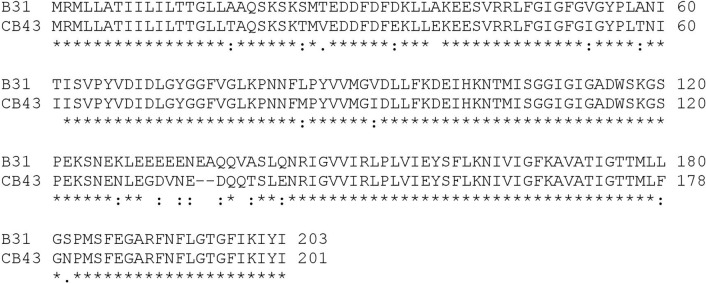
Alignment between the BB0405 protein sequence between *Borrelia burgdorferi* B31 and Borrelia afzelii PKO. *Borrelia burgdorferi* B31 corresponds to NCBI Reference Sequence: NP_212539. Borrelia afzelii PKO is identical to CB43 (GenBank: CP002933.1 translated in ExPASy translate tool). Identity between sequences is 88.2%. Additionally there are 17 similar positions bringing the similarity to 96.5%. Alignment was performed with Clustal Omega. * = identical, : = similar.

**Figure 2:**
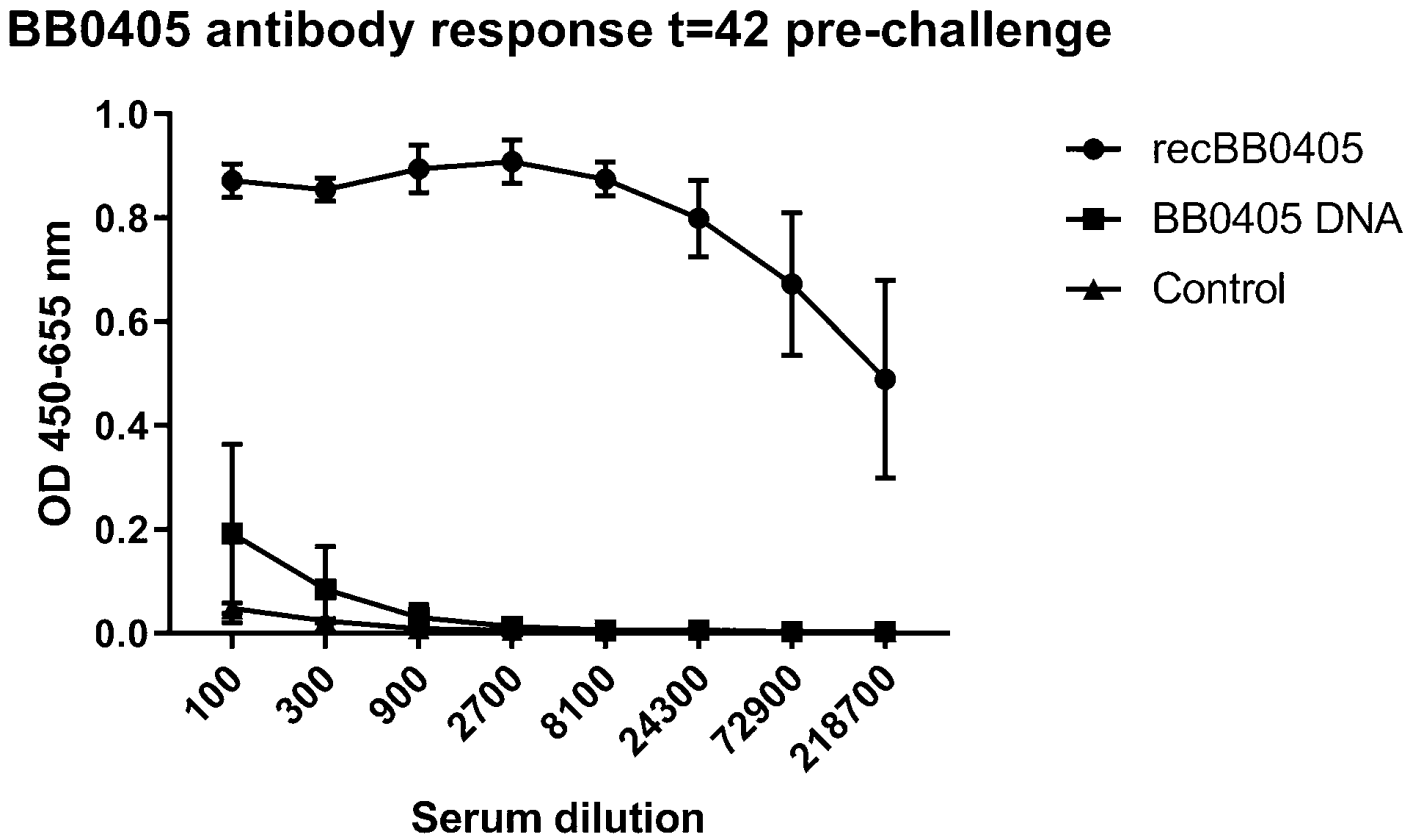
Enzyme-linked immunosorbent assay (ELISA) showing high-titer antibodies induced in recombinant BB0405 immunized mice. BB0405 specific total IgG responses were measured in mice sera of three vaccination groups with 8 mice each. IgG responses are presented as optical density (OD) 450–655 nm for multiple sera dilutions. The first group was immunized at three time points (0, 14 and 28 days) with recBB0405. The second groups received DNA vaccinations at the same time points with DNA BB0405 and the third group with DNA Empty (negative control). Plates were coated with recombinant BB0405 and incubated with mouse sera, which was collected from mice at time point 42 days, just before tick challenge and after three vaccinations. Sera was diluted in steps of 300 until 1: 218700.

As described previously, BB0405 is a highly conserved *B. burgdorferi* sl surface protein and alignment of the protein *BB0405* of *B. burgdorferi* B31 and *B. afzelii* CB43 showed an identity of 88% and similarity of 96% at the amino acid sequence level (Fig. 1). To determine whether antibodies against BB0405 would protect across different *B. burgdorferi* sl genospecies, we performed a vaccination study in mice. Recombinant BB0405 and a DNA vaccine for *bb0405*, both generated from *B. burgdorferi* B31, were constructed as well as an empty DNA vaccine, functioning as the negative control. From our previous published and unpublished studies it is known that an empty DNA vaccine, i.e. a pVAX vector without inserted target sequences, does not affect *B. burgdorferi* sl infection (^10^ and Klouwens et al. manuscript in preparation). Two weeks after the 3rd vaccination, mice were challenged with *B. afzelii* CB43-infected *Ixodes ricinus* nymphs. To assess immunogenicity, BB0405 specific total IgG levels were measured by ELISA before and after vaccinations (Fig. 2). As expected, the BB0405 specific total IgG levels were very high in the mice that had received the recombinant BB0405 vaccine. In contrast, this was not the case in the mice that had received the *bb0405* DNA vaccine. In fact, the antibody titers in this group were comparable to the control mice that had been vaccinated with the empty DNA vaccine.

### Host-protection via BB0405 immunization

Next, we assessed whether both vaccines were able to provide protection against *B. afzelii* infection by performing qPCR and culture of several tissues, obtained by sacrificing the mice 21 days after challenge with *B. afzelii* strain CB43-infected ticks. *B. afzelii* loads in ear, skin (tick feeding site), heart, bladder and ankle were quantified and normalized to mouse *β-actin*. In addition, bladder and skin tissues from the feeding site of the ticks were cultured and checked weekly for the growth of spirochetes. Despite the high antibody titers, results of qPCR and culture indicate that vaccination with BB0405, either recombinant or as DNA vaccine, did not protect against heterologous *Borrelia* challenge in mice (Table 1). Indeed, there were no significant differences between the separate groups in the different tissues, nor between experimental groups at a cumulative level.

**Table 1:** Overview of number of Borrelia positive mice per group in qPCR tissues and culture.

Vaccin	qPCR positivity					Culture positivity		Cumulative positivity
	Skin	Ear	Bladder	Heart	Ankle	Skin	Bladder	
Rec BB0405	4/8	6/8	2/8	4/8	6/8	4/8	4/8	8/8
DNA BB0405	3/8	4/8	1/8	2/8	4/8	2/8	2/8	5/8
Control	5/8	4/8	4/8	3/8	3/8	3/8	2/8	8/8

Each experimental group consisted of 8 mice. qPCR was performed on 5 different tissues: skin (tick bite site on the back), ear, bladder, heart and ankle. Both skin and half of the bladder were cultured in MKP medium and checked for growth of Borrelia for 8 weeks. Statistical significance of cumulative data of the experimental groups compared to the DNA Empty was calculated with the Fisher’s exact test. Comparison between all separate groups was not statistically significant.

### BB0405 surface expression *B. burgdorferi* sensu stricto and *B. afzelii*

**Figure 3:**
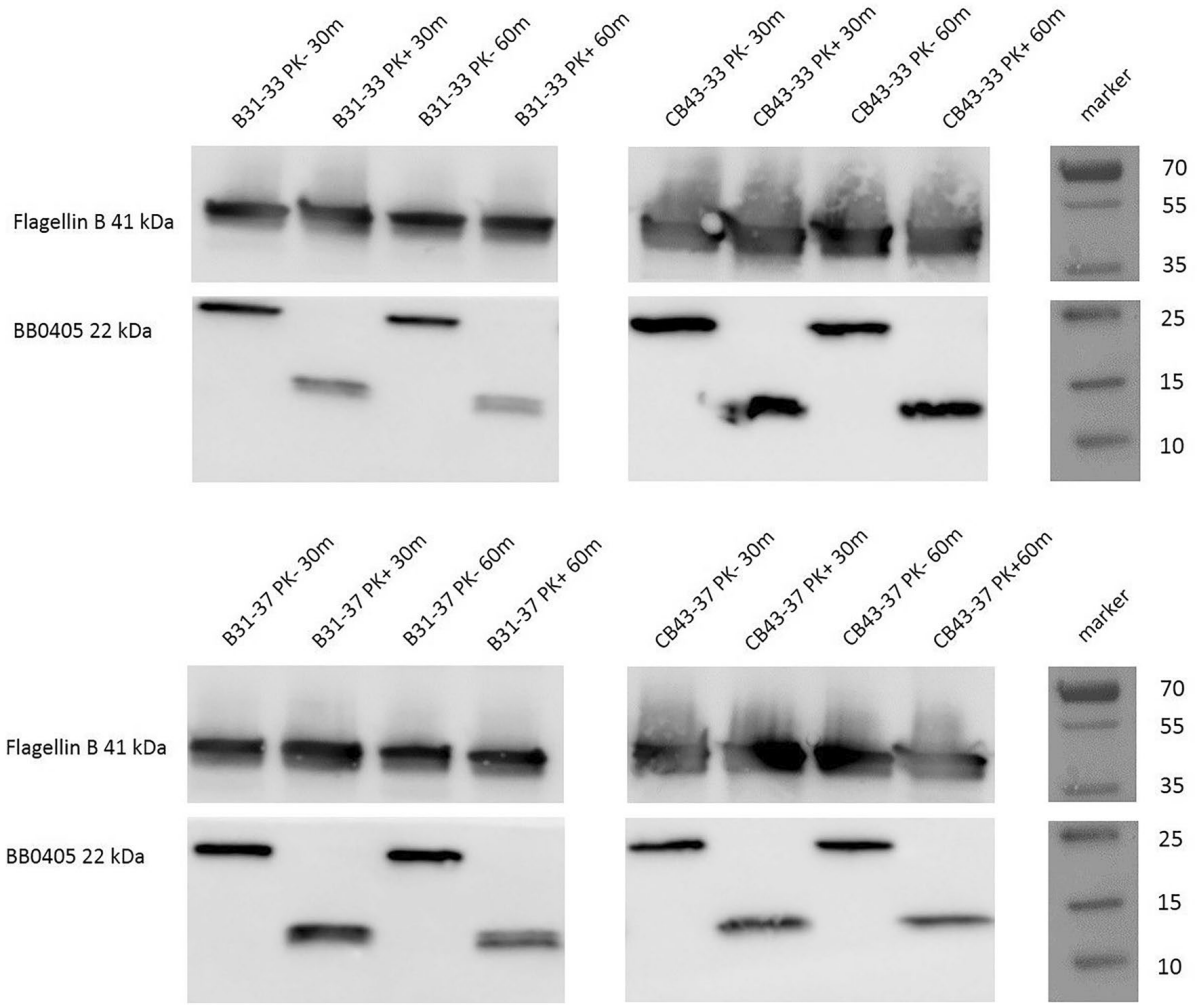
BB0405 is expressed on the surface of *B. burgdorferi* sensu stricto and *B. afzelii*. Viable spirochetes were incubated with (+) or without (−) proteinase K for 30 min and 60 min and processed for immunoblot analyses with antibodies against BB0405. Flagellin B antibodies served as a subsurface control.

To study the location of the protein and also the potential accessibility of the protein to antibodies we performed a proteinase K assay with viable *B. burgdorferi* ss and *B. afzelii* spirochetes followed by Western Blot analysis. We show that BB0405 is partly surface exposed as the protein is partly digested upon proteinase K treatment for both 30 and 60 min both in *B. burgdorferi* ss as well as in *B. afzelii. *Flagellin B, a periplasmic control protein, was, as expected, not affected by proteinase K treatment (Fig. 3).

### Expression of BB0405 or homologous proteins in *B. burgdorferi* sensu stricto and *B. afzelii*

**Figure 4:**
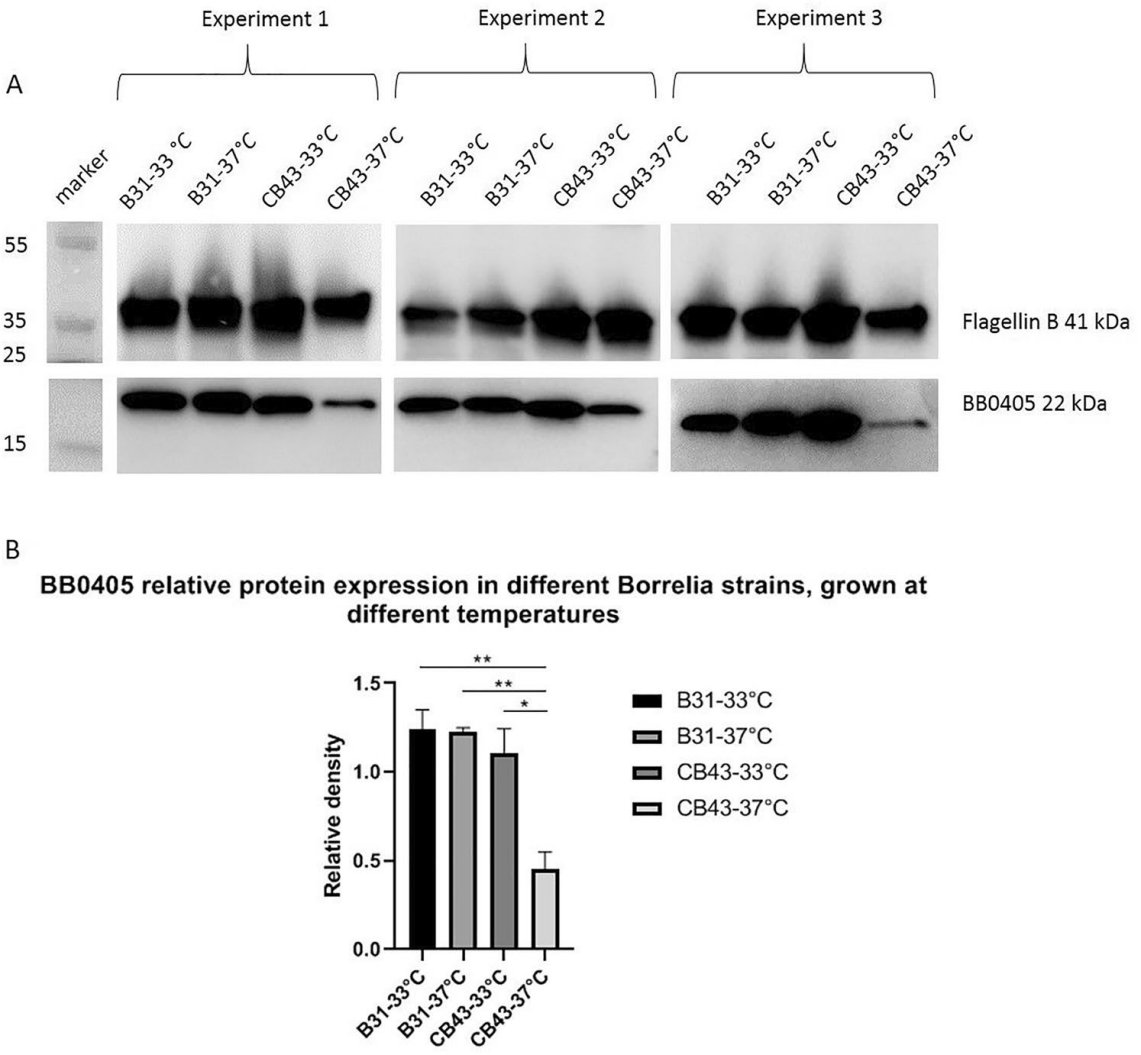
(**A**) Western blot showing expression of BB0405 in different *B. burgdorferi* sl strains (*Borrelia burgdorferi* strain B31 and *Borrelia afzelii* strain CB43) grown at different temperatures. A temperature of 33 °C, resembling (feeding) tick conditions, and 37 °C, resembling conditions in the mammalian host, was used to culture the spirochetes. Lysates of the in vitro cultured spirochetes were obtained, as described in the materials and method section, and subjected to Western blot. Three independent experiments were performed. The first experiment is displayed on the left, the second experiment in the middle and the third experiment is displayed on the right. The far left lane is the protein weight marker. Blots were loaded with 2.5 ug/well whole lysates of spirochetes and were cut in half just between the bands of Flagellin B (41 kDa) and BB0405 (22 kDa) to enable separate incubations. The blots displayed at the top are incubated with anti-flagellin rabbit IgG 1:1000 (as a loading control) and subsequently with secondary antibody anti-rabbit IgG-HRP 1:1000. Blots displayed at the bottom are incubated with pooled serum from mice vaccinated with recombinant BB0405 1:500 and subsequently with secondary antibody anti-mouse IgG-HRP 1:2000, respectively. Blot images were cropped (Image acquisition tools Microsoft Powerpoint). Imaging was performed using ImageQuant LAS 4000 and quantification using Image J (Wayne Rasband, National Institutes of Health, USA, Java 1.8.0_77(32-bit), http://imagej.nih.gov/ij). Full length blots are presented in Supplementary Fig. S1–S6. (**B**) Protein expression of BB0405 as determined by Western blot in panel A was quantified and normalized against the relative density of loading control Flagellin B. Relative density was determined using ImageJ software (National Institute of Health). A quantitative comparison between samples on the same blot and within the same experiment were made. Statistical differences between groups were calculated using an unpaired parametric t-test. Error bars represent mean ± s.e.m. n.s.: *P* > 0.05.

Finally, in an attempt to understand why, in contrast to previous observations for *B. burgdorferi* ss^7,8^, BB0405 did not protect against *B. afzelii*, we next precisely compared the expression levels of BB0405 between these two different *B. burgdorferi* sl genospecies under different experimental conditions. We did not find differences in expression of *bb0405* at the RNA level between *B. burgdorferi* ss and *B. afzelii* grown at 33 °C or 37 °C (data not shown). However, we also assessed BB0405 expression at the protein level. To this end, equal amounts of whole lysates of spirochetes, cultivated in vitro at different temperatures, were run on a Western blot and incubated with BB0405 specific antibodies. As can be appreciated from Fig. 4A, B*. afzelii* CB43 produces considerably lower amounts of BB0405 at 37 °C, the condition similar to the conditions in the mammalian host. We also quantified the protein bands on the Western Blots (Image J software) and measured the relative density of the bands compared to a corresponding Flagellin B loading control (Fig. 4B). Indeed, the relative density of BB0405 in *B. afzelli* CB43 cultured at 37 °C was significantly lower compared to BB0405 expression at 33 °C and to *B. burgdorferi* B31 at 37 °C, offering a possible explanation for our findings.

## Discussion

An ideal Lyme vaccine candidate would need to provide protection against the most dominant *B. burgdorferi* sl genospecies causing Lyme borreliosis. The main *B. burgdorferi* sl genospecies causing Lyme borreliosis in North-America is *B. burgdorferi*ss, whereas in Europe this is *B. afzelii*. To explore the potential of BB0405, in the current study we investigated whether vaccination with BB0405—a surface-exposed *B. burgdorferi* ss protein that is well-conserved among different *B. burgdorferi* sl genospecies including *B. afzelii*—protected against heterologous challenge with *B. afzelii* through tick-bite in an experimental mouse model. Moreover, two different methods of vaccination with BB0405 were used, recombinant protein vaccination and DNA vaccination by tattoo. Finally, to provide further insights into our findings we assessed the differential BB0405 expression of *B. burgdorferi* ss and *B. afzelii* under varying conditions.

An adequate humoral response is essential in clearing *Borrelia* spirochetes and specific immunoglobulins play a key role in affording protective host immunity^11–13^. Since BB0405 is an outer surface protein, it is surface exposed and therefore likely to be antibody-accessible. Surprisingly, Kung et al. described the interesting phenomenon that no BB0405 specific antibodies are generated during natural infection in mice; however Brooks et al. showed that recombinant BB0405 is immunogenic in rats and antibodies raised against BB0405 could kill *B. burgdorferi ss *in vitro in the presence of complement^6^. In addition, in mice immunized with recombinant BB0405 a strong and long-lasting antibody response was induced that provided protective immunity against tick-transmitted infection with *B. burgdorferi*^8^. In the current study we also show a robust humoral immune response in mice vaccinated with recombinant BB0405 compared to control mice, corroborating the observation that recombinant BB0405 is immunogenic. In contrast, we did not observe antibody responses against BB0405 in the mice vaccinated with the *bb0405* DNA vaccine. Interestingly, the same DNA vaccination approach has been successful in the past for other *Borrelia* outer surface proteins^10^. In our study this could indicate that the mice cells were not able to transcribe, translate or translocate *bb0405* and that the utility of DNA vaccination against *B. burgdorferi* sl is highly dependent on the target. However, as murine infection with *B. burgdorferi* ss*,* either by syringe or ticks, does not result in BB0405-specific antibody responses, it could also be that *bb0405* needs the adjuvants used in recombinant protein vaccinations and is not immunogenic by itself^8^.

In the study described here, we have challenged the BB0405 (*B. burgdorferi B31* derived)-immunized mice with *B. afzelii* infected ticks. We did not observe any protection of BB0405, neither as recombinant vaccine nor as DNA tattoo. Based on our observations, BB0405 does not seem to be a suitable vaccine candidate for the European situation, as it does not provide cross-protection between *B. burgdorferi* sl species—in this case *B. burgdorferi* ss and *B. afzelii*—despite high antibody titers after vaccination with recombinant protein. Although we have performed a heterologous challenge, the in silico analysis showed high sequence homology between these proteins. Indeed in Figs. 3 and 4 we show that the raised antibodies recognize and bind to BB0405 expressed by *B. afzelii*. It seems therefore unlikely that antigen recognition can explain the observed lack of vaccine efficacy. Future studies could assess whether using different species-specific BB0405 homologues from different *B. burgdorferi* sl genospecies could protect against heterologous challenge.

As stated above, antigen specificity of the generated antibodies is unlikely to explain the observed lack of efficacy. Therefore we firstly wanted to determine whether BB0405 was indeed surface expressed in *B. afzelii* strain CB43, as has been shown *B. burgdorferi* ss strain B31^8^. We have shown that BB0405 is indeed partly expressed at the cell surface by performing a Western Blot analysis after treating viable *B afzelii* B43 spirochetes with proteinase K. We used *B. burgdorferi* B31 as a control. The reason that there is only partial surface expression in both *B. burgdorferi* sl strains can be explained by BB0405 being a transmembrane protein and thus partly intracellular or membrane bound, explaining the lower protein products on the Western Blots of Fig. 3. Secondly, there are over 20 different species within the *B. burgdorferi* sl complex and these are capable of molecular adaptations in order to ensure efficient transmission by their vector and to survive in different environments of their hosts^14–18^. Temperature is a key environmental factor known to affect *B. burgdorferi* sl gene expression. More specifically, it has been described by Ojaimi et al. that BB0405 is upregulated by temperature suggesting upregulation in conditions that mimic the situation in the mammalian host^17^. We therefore wondered whether a difference in protein expression of BB0405 between American and European *B. burgdorferi* sl genospecies could explain the lack of protection in our vaccination experiment. At the protein level we observed a distinct and significantly lower expression of BB0405 in *B. afzelii* CB43 spirochetes grown at 37 °C, as compared to the expression in the same spirochetes grown at 33 °C. More importantly, expression of BB0405 in *B. afzelii* CB43 spirochetes grown at 37 °C was also significantly lower compared to *B. burgdorferi* B31 spirochetes grown at the same temperatures. This difference was not apparent at the RNA level. However, it is well-known that RNA expression levels do not necessarily correspond to the protein expression levels^19^. Thus, the BB0405 protein seems to be evidently less expressed by *B. afzelii* CB43 in conditions reflecting the mammalian host and this might explain the observed lack of protection against *B. afzelii* strain CB43, despite the presence of specific antibodies upon vaccination with recombinant BB0405. An alternative explanation for the lack of protection could be an indirect effect of failure of antibody recognition. *B. burgdorferi* sl is able to alter surface protein in vivo via differential gene expression and via VlsE recombination which might impact spirochete recognition by host-generated antibodies^20–23^. There are multiple examples of surface-exposed Borrelial antigens that are masked by neighboring (abundant) proteins^15,24^. Although BB0405 is conserved across *B. burgdorferi* sl genospecies, it could be possible that expression of other surface proteins is different in *B. burgdorferi* ss and *B. afzelii,* which may account for different surface topology and outcome of antibody-mediated protections.

In conclusion, being a surface-exposed, immunogenic and well-conserved *B. burgdorferi* sl protein, BB0405 was shown to be an interesting vaccine candidate to protect against Lyme borreliosis caused by *B. burgdorferi* ss. However, we here show that vaccination with *B. burgdorferi* ss-derived BB0405 does not protect against heterologous challenge with *B. afzelii* through tick-bite, and our data suggest that this could be due to lower expression of the BB0405 homologue at the protein level in *B. afzelii* in the mammalian host. Nevertheless, experiences with the OspA vaccine have shown that a vaccine does not have to protect against all *B. burgdorferi* sl genospecies to be commercially viable. Future experiments should investigate whether multivalent BB0405 vaccines are able to protect against multiple *B. burgdorferi* sl genospecies.

## Methods

### Ethics statement

All experiments were reviewed and approved by the Animal Research Ethics Committee of the Academic Medical Center, Amsterdam, The Netherlands (protocol 208AK-1 and 271AA-1). Experiments have been conducted according to European and national guidelines http://eur-lex.europa.eu/legal-content/EN/TXT/?uri=celex:32010L0063 ) and in compliance with the ARRIVE guidelines (http://www.nc3rs.org.uk/page.asp?id=1357).

### Recombinant BB0405 protein

The *bb0405* gene was cloned without the N-terminal leader sequence into pET28a (Invitrogen), produced in *E. coli*, and purified using Ni–NTA resin as detailed elsewhere^8^. Sequence alignment between BB0405 from different strains was performed using Clustal Omega^25^.

### Generation of the BB0405 DNA vaccine

The pVAXhTPA-BB0405 DNA vaccine was designed as described before in Wagemakers et al.^10^. In the BB0405 gene sequence of *B. burgdorferi* B31 (NCBI Reference Sequence: NC_001318.1) the 18aa signal sequence (predicted by SignalP 4.0 web-based software, CBS, Lyngby, Denmark) was replaced with the human tissue plasminogen activator (hTPA) signal sequence (genbank AAA61213.1)^26^. The resulting sequence was codon-optimized to mouse tRNA usage with Java Codon Adaptation tool (Braunschweig, Germany)^27^. At the 5′ end a BamH1 and a Kozak sequence were added, and at the 3′ end a sequence encoding a double stop codon and a Xho1 were added. The insert was synthesized (BaseClear, Leiden, The Netherlands) and ligated into a BamH1/Xho1 restricted empty pVAX vector (Invitrogen, Carlsbad, CA, USA). The plasmid was amplified using a Nucleobond Xtra EF kit (Macherey-Nagel, Düren, Germany) and resuspended in DNase free water.

### Vaccination experiments

**Figure 5:**
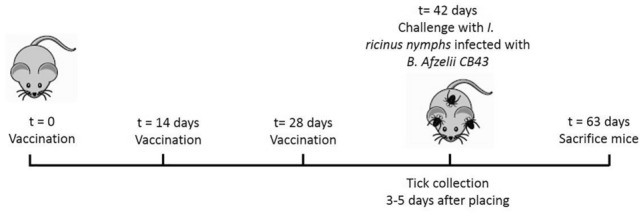
Schematic design of the vaccination study. The study consisted of 3 experimental groups of 8 mice each. The first group was vaccinated with recombinant BB0405, the second group was received a tattooed DNA vaccination with BB0405 and the third group received an empty vector DNA vaccination as a control. Mice were vaccinated at t = 0, t = 14 and t = 28 days. They were challenged with infected ticks 2 weeks after the last booster vaccination and sacrificed at day 63.

Six to eight weeks old female C3H/HeN mice were purchased from Charles River and the experiment was designed with 3 groups of eight mice each. The vaccination experiment was carried out as described in a previous publication of our group^10^. The first group was vaccinated with a recombinant BB0405 vaccine (recBB0405), the second group with a BB0405 DNA vaccine, and the third group consisted of an empty vector DNA vaccination group as a negative control. Mice were vaccinated at t = 0, t = 14 and t = 28 days and sera were collected at each time point for use in ELISA experiments. For the recombinant BB0405 vaccine 10 μg protein was emulsified with complete Freund’s adjuvant at t = 0 and 5 μg in incomplete Freund’s adjuvant at t = 14 and t = 28 days (Fig. 5). All vaccinations were administered subcutaneously. For the pVAX-hTPA-BB0405 DNA vaccine and the negative control hair was removed from the mice abdomens using hair removal cream (Veet). Using a Cheyenne Hawk tattoo machine carrying a Cheyenne 13-magnum tattoo needle (both MT.DERM, Berlin, Germany) 20 μg of the DNA vaccines was tattooed 0.5–1 mm into the abdominal skin of the mice for 45 s at 100 Hz under isofluorane anesthesia. Two weeks after the third vaccination, at t = 42, all mice were challenged with 5 *Ixodes ricinus* nymphs infected with *Borrelia afzelii* strain CB43. These *B. afzelii*-infected *I. ricinus* nymphs were placed in a containing capsule and fed on the immunized and control animals until repletion. Additional sera were collected at t = 42 (pre-challenge, 2 weeks after the third immunization) and at t = 63 days (3 weeks post-challenge) mice were sacrificed and ear, skin, ankle, heart, bladder and tissue was collected for analysis. Mice were considered to be infected when there was at least one positive tissue sample in either qPCR or culture.

### ELISA

To measure IgG directed against BB0405, ELISAs were performed according to previous described protocol^10^. High-binding 96-well ELISA plates (Greiner Bio-one, Kremsmünster, Austria) were coated overnight at 4 °C with 1 μg/ml recBB0405, washed with PBS–Tween (phosphate-buffered saline–0.05% Tween) and incubated with blocking buffer (1% BSA in PBS) for 2 h at room temperature. Mouse sera (collected at day 42 before tick challenge) were diluted in blocking buffer, added to the wells and incubated for 1 h at room temperature. Plates were washed and incubated for 1 h with horseradish peroxidase (HRP)-linked anti-mouse IgG (Cell Signaling, Beverly, MA, USA) diluted 1:1000 in blocking buffer. The plates were washed again and developed using TMB substrate (50 μl TMB chromogene in 5 ml TMB substrate buffer (8.2 g NaAc and 21 gr citric acid monohydrate dissolved in 1 L H_2_O + 10 μl 3% H_2_O_2_) and optical density was measured in a Biotek (Winooski, VT, USA) ELISA plate reader at 450–655 nm.

### *Borrelia afzelii* detection and quantification

Murine bladder and skin samples were cultured in modified Kelly Pettenkofer (MKP) medium with rifampicin, 50 µg/ml and phosphomycin, 100 µg/ml) at 33 °C. The cultures were checked weekly (for a total of 8 weeks) for the presence of motile spirochetes with dark field microscopy as described before^28^. For all samples DNA was extracted using Qiagen Blood and Tissue kit (Qiagen, Venlo, The Netherlands). Quantitative (q)PCR was used to quantify *B. afzelii* DNA in mouse tissues and was performed according to previously described protocol^10^. OspA primers were used for quantification; forward 5′-AAAAATATTTATTGGGAATAGGTCT-3′ and reverse 5′-CACCAGGCAAATCTACTGAA-3′, mouse Beta-actin forward 5′-AGCGGGAAATCGTGCGTG-3′ and reverse primer 5′-CAGGGTACATGGTGGTGCC-3′ were used for normalization. The qPCRs were performed on the LightCycler480 (Roche, Nutley, NJ, USA) using SYBR green dye (Roche) using the following PCR protocol: 95 °C 6 min, and 60 cycles of 95 °C 10 s, 60 °C 20 s and 72 °C 20 s. Reactions were performed in triplicate. Results were analyzed using LinRegPCR software (Amsterdam, The Netherlands)^29^. Negative and positive controls were included in each qPCR run. A positive *Borrelia* load was determined by at least one melting curve exactly matching the positive control (skin tissue known to be infected with *B. afzelii*). In case of a negative value for OspA in a sample, the OspA value was replaced by the value of the OspA detection limit. The detection limit is defined as the highest dilution of the control sample in which OspA is still detectable + 3xSD.

### RNA and cDNA synthesis

*B. burgdorferi* B31 and *B. afzelii* CB43 were cultured in MKP medium at 33 °C or 37 °C to 1 × 10e6 spirochetes/ ml as assessed by a Petroff-Hausser counting chamber and dark-field microscopy. The cultures were centrifuged for 10 min at 10.000 rpm and the pellets were dissolved in 750 µl RNA Later (Qiagen) stored at − 80 °C until further use. All samples were thawed simultaneously and centrifuged for 5 min at 12.000 rpm. The pellets were then subsequently used for isolation of RNA using the Nucleospin RNA isolation kit (Macherey–Nagel), according to the manufacturer’s instructions. Subsequently, RNA samples were digested by DNase for a second time using the Qiagen RNase-Free DNase Set (#79254) and then cleaned up using the RNeasy MinElute Cleanup Kit (#74104, Qiagen). A total of 10 µl of each RNA sample was then used to generate cDNA and heated for 5 min at 85 °C and then cooled to 23 °C. To every sample, 10 µl of RT mix was added, consisting of 4 µl of M-MLV reverse transcriptase buffer (Promega), 0.5 µl of M-MLV reverse transcriptase enzyme 200U/ml (Promega), 2 µl dNTP mix (Invitrogen), 1 µl of random hexamers (), 0.5 µl DTT (Invitrogen), 0.5 µl RNaseOut (Invitrogen), as well as 1.5 µl of RNase free H2O. The PCR protocol for all samples was 23 °C 10 min, 42 °C 60 min and 95 °C 3 min (2720 Thermal Cycler, Applied Biosystems). To check the purity of the RNA, a PCR was performed using RNA and cDNA (100× diluted) of corresponding samples as a template with universal *flaB* primers (*flaB* forward 5′-GCTTCTGATGATGCTGCTG-3′ and *flaB* reverse 5′-CGTCTGTAAGTTGCTCTATTTC-3′) and PCR Phusion High Fidelity Mastermix (#M0531S, NEB). As expected, there was no amplification in the samples were RNA was used as template, indicating there was no DNA contamination. Real-time quantitative polymerase chain reaction (qRT-PCR) was used to quantify the amount of BB0405 RNA in the two different *B. burgdorferi* sl strains, grown at different temperatures. The qRT-PCR was performed with BB0405 specific primers for *B. afzelii* CB43 (forward 5′- GTCTGTGCGCCGTTTATTTG-3′ and reverse 5′-GGGTTTAAGTCCAACGAATCC-3′) and for *B. burgdorferi* B31 (forward 5′-GGCATAGGTTTTGGAGTTGG-3′ and reverse 5′-CCATCACAACATAGGGCAAG-3′) and with Flagellin B universal primers as housekeeping genes (forward 5′-GCTTCTGATGATGCTGCTG-3′ and reverse 5′-CGTCTGTAAGTTGCTCTATTTC-3′). The cDNA from the two different *B. burgdorferi* sl strains grown under different conditions was diluted 100 times and 1 µl was used as template per reaction and qPCRs were performed using the LightCycler480 (Roche, Nutley, NJ, USA) and SYBR green dye (Roche) in triplicate. The PCR protocol was 95 °C 6 min, and 60 cycles of 95 °C 10 s, 60 °C 20 s and 72 °C 20 s. Results were analyzed using LinRegPCR software (Amsterdam, The Netherlands).

### Borrelia lysates and BB0405 western blots

*B. burgdorferi* B31 and *B. afzelii* CB43 were cultured in MKP medium at 33 °C or 37 °C until log phase was reached, after which the cultures were centrifuged and the spirochete pellets were washed three times in PBS for 10 min at 10.000 g. Ultimately, the pellets were dissolved in 250 µl of PBS and were sonicated on ice (6 times 15 s on, 30 s off with 20% amplitude using Vibra-Cell High Intensity ultrasonic processor). Protein concentrations were measured using the Pierce BCA protein assay (#23225 from Thermo Scientific). Subsequently, lysates (2.5 µg protein per sample) were mixed in a 5:1 ratio with 5 × SDS sample reducing buffer (1.5% SDS, 10% glycerol, 62.5 mM Tris–HCl (Ph 6.8), 2%-mercapto-ethanol and 0.0025% bromophenol blue) and subjected to sodium dodecyl sulfate–polyacrylamide gel electrophoresis (SDS-PAGE) using a precast SDS 4–20% polyacrylamide gel (Bio-Rad Mini-PROTEAN TGX) with PageRuler Plus Prestained Protein Ladder (Thermo Scientific). Samples were then transferred to a 99% ethanol-activated PVDF membrane. After blotting, the membrane was cut in half just between the bands of Flagellin B (41 kDa) and BB0405 (22 kDa) to enable separate incubations. The upper half of the membrane, containing Flagellin B, was incubated with a 1:1.000 dilution of anti-flagellin rabbit IgG (Anti-Flagellin RABBIT Antibody, #200-401-C14S, Rockland) as a loading control. The lower half of the membrane, containing BB0405, was incubated with a 1:500 dilution of murine sera derived from mice immunized with recombinant BB0405 (*B. burgdorferi* B31). Immunoblots were subsequently labeled with HRP-conjugated secondary antibodies (anti-rabbit IgG-HRP 1:1000 and anti-mouse IgG-HRP 1:2000 respectively, by Cell Signaling) and developed using Pierce ECL Western Blotting Substrate (#32106 Thermo scientific). Imaging was performed using ImageQuant LAS 4000 and quantification using Image J (Wayne Rasband, National Institutes of Health, USA, Java 1.8.0_77(32-bit), http://imagej.nih.gov/ij).

### Proteinase K assay

Proteinase K assays were carried out as described elsewhere^30–32^. Spirochetes in mid-phase growth were washed twice with PBS + 5 mM MgCl_2_ and were pelleted. Pellets were resuspended gently and split in equal volumes. They were incubated in PBS + 5 mM MgCl_2_ either in the absence or presence of proteinase K at a concentration of 200 µg/ml for either 30 or 60 min at room temperature. Phenylmethylsulfonyl floride (PMSF)(Sigma) was added (1.5 mM) to stop proteinase K activity. Spirochetes were again washed twice in PBS + 5 mM MgCl_2_ + 1 mM PMSF and pelleted. The pellets were resuspended in an appropriate volume of SDS-PAGE loading buffer for further Western Blot analysis. Western Blots were carried out as described above.

### Statistic methods

Differences between experimental groups between Borrelia loads in qPCR were statistically tested by two-sided nonparametric tests (Mann–Whitney, GraphPad Prism software version 5.0, San Diego, CA, USA). Differences in expression level between experimental groups on Western Blot were compared using an unpaired t test (Graphpad Prism software version 5.0, San Diego, CA, USA).

## Supplementary Information

Supplementary Figures.
