# Supplementary material for: Investigating BB0405 as a novel Borrelia afzelii vaccination candidate in Lyme borreliosis
Source: Sci Rep. 2021 Feb 26;11:4775. doi: 10.1038/s41598-021-84130-y (PMC7910573; doi:10.1038/s41598-021-84130-y)
Supplement: Supplementary file 1 — Supplementary Figures. [file 41598_2021_84130_MOESM1_ESM.docx]

**Supplementary Information**

**Investigating BB0405 as a novel *B. afzelii* vaccination candidate in Lyme Borreliosis**

**Authors**

^*^Klouwens MJ^1,2,3^, Trentelman JJ^1^, Ersoz JI^1^, Nieves Marques Porto F^1^, Sima R^4^, Hajdusek O^4^, Thakur M^5^ , Pal U^5^ , Hovius JW^1,2,3^

**Affiliations**

^1^Department of Internal Medicine, Center for Experimental and Molecular Medicine, Academic Medical Center, University of Amsterdam, Amsterdam, The Netherlands.

^2^Division of Infectious Diseases, Department of Internal Medicine, Academic Medical Center, Amsterdam, The Netherlands.

^3^Amsterdam Multidisciplinary Lyme borreliosis Center, Academic Medical Center, Amsterdam, The Netherlands.

^4^ Biology Centre, Institute of Parasitology, Czech Academy of Sciences, Ceske Budejovice, Czech Republic.

^5^ Department of Veterinary Medicine, University of Maryland, College Park and Virginia- Maryland Regional College of Veterinary Medicine, College Park, Maryland, USA.

**Corresponding author**

^*^Klouwens MJ, Department of Internal Medicine,

Center for Experimental and Molecular Medicine, Academic Medical Center, University of Amsterdam, Meibergdreef 9, Amsterdam 1105 AZ, The Netherlands.

E-mail: [m.j.klouwens@amsterdamumc.nl](mailto:m.j.klouwens@amsterdamumc.nl)

*Supplemental Figure S1. Experiment 1 Flagellin B, full length Western blot, uncropped*


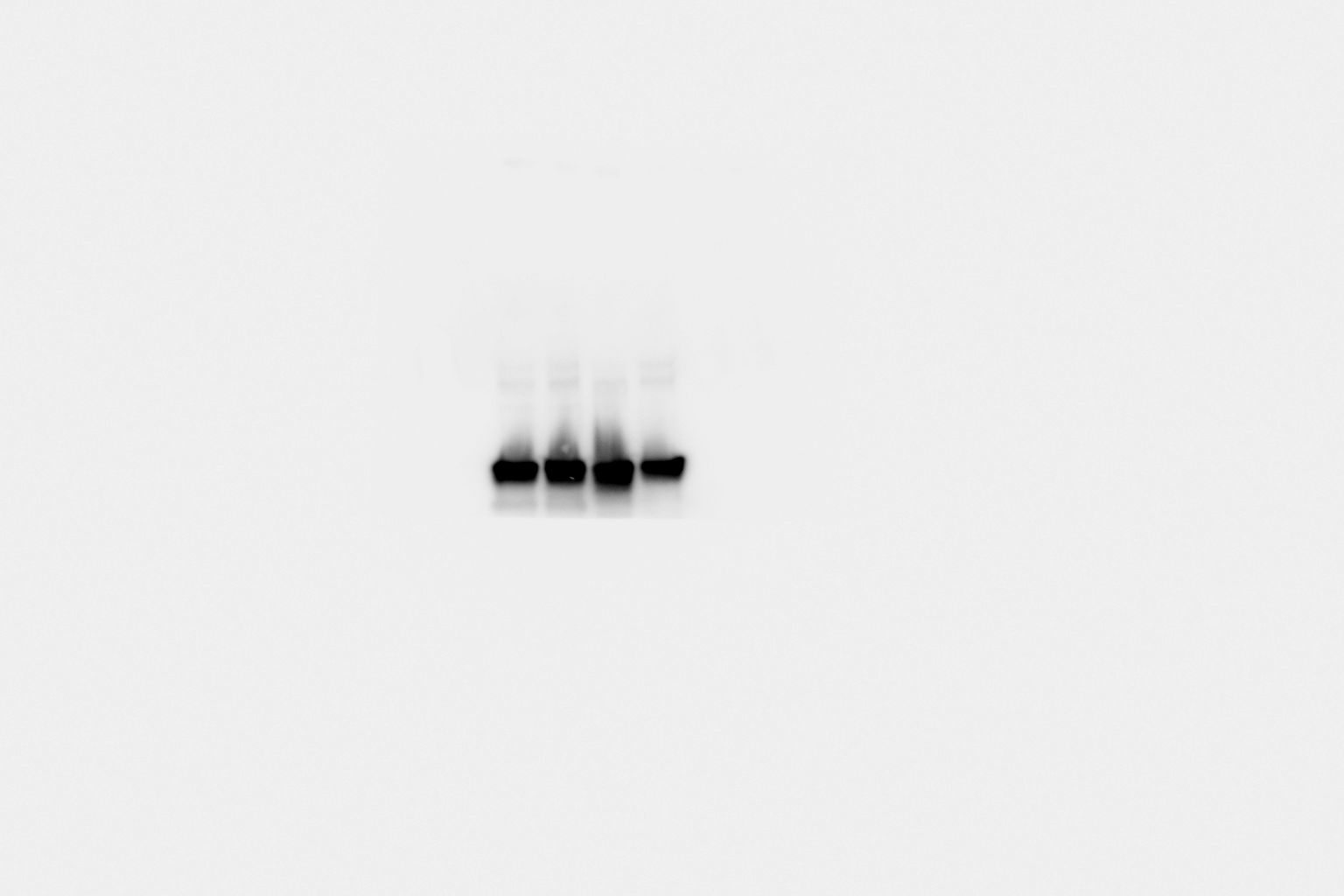


*Supplemental Figure S2. Experiment 1 BB0405, full length Western blot, uncropped*

**
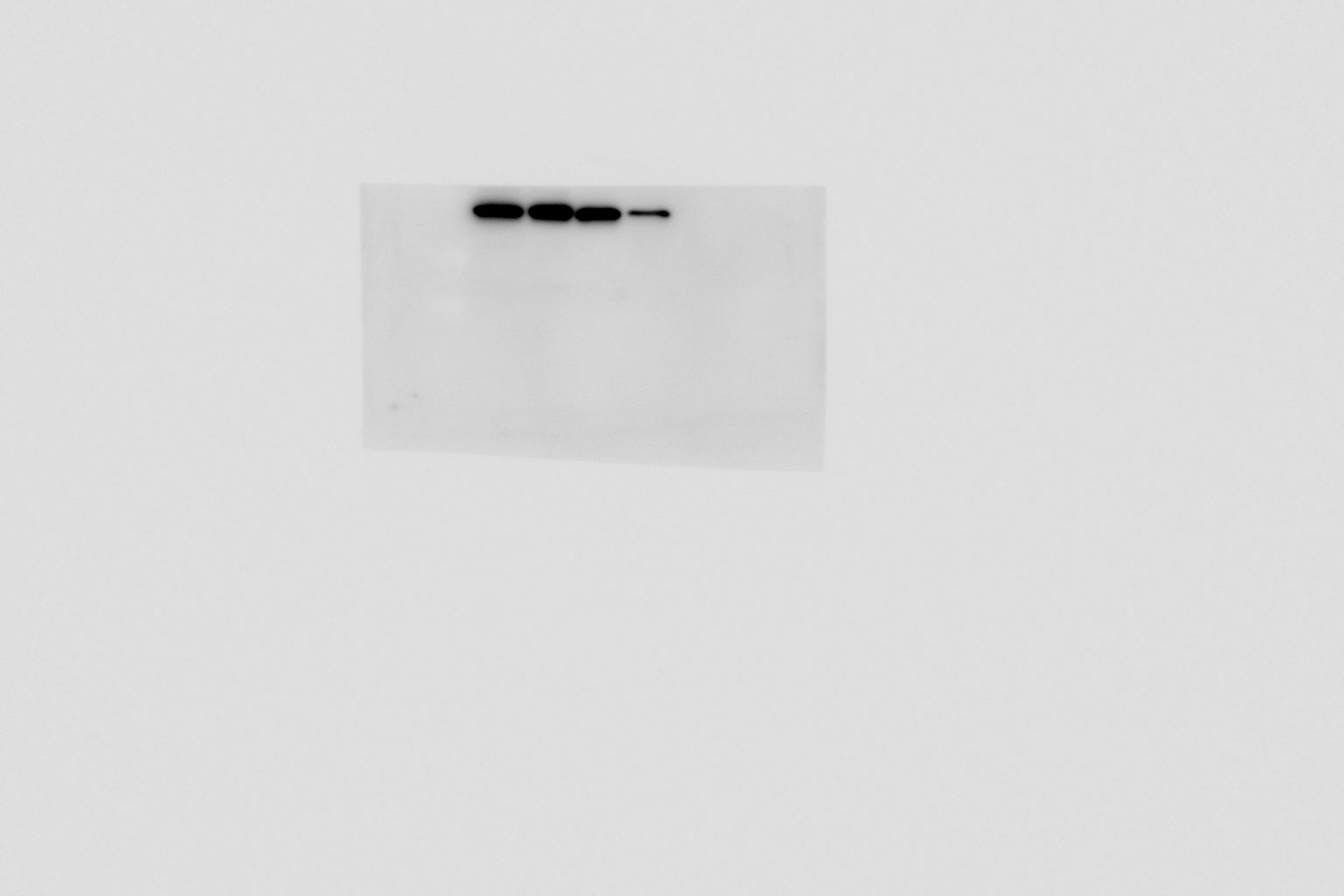
**

*Supplemental Figure S3. Experiment 2 Flagellin B, full length Western blot, uncropped*

**
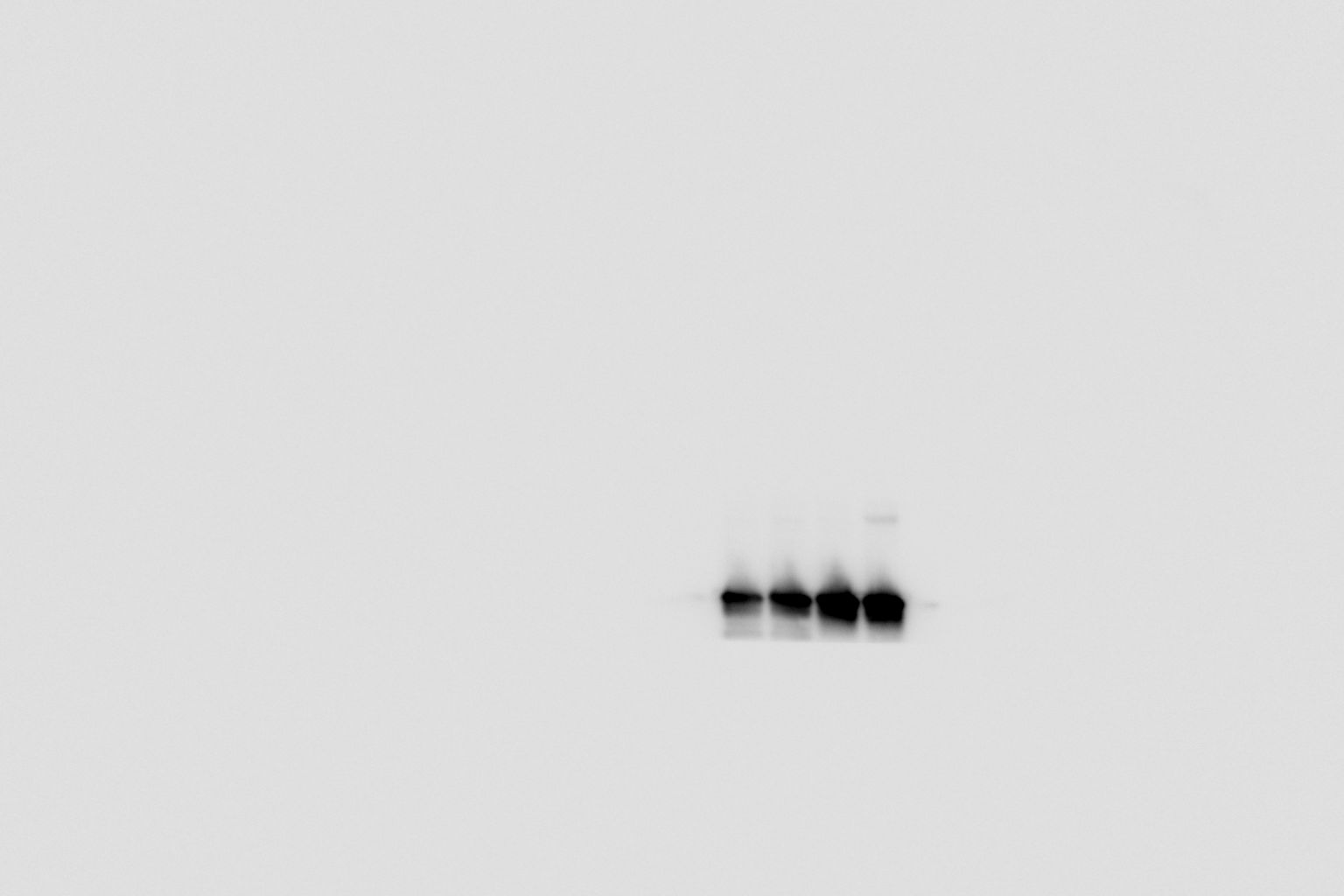
**

*Supplemental Figure S4. Experiment 2 BB0405, full length Western blot, uncropped*

**
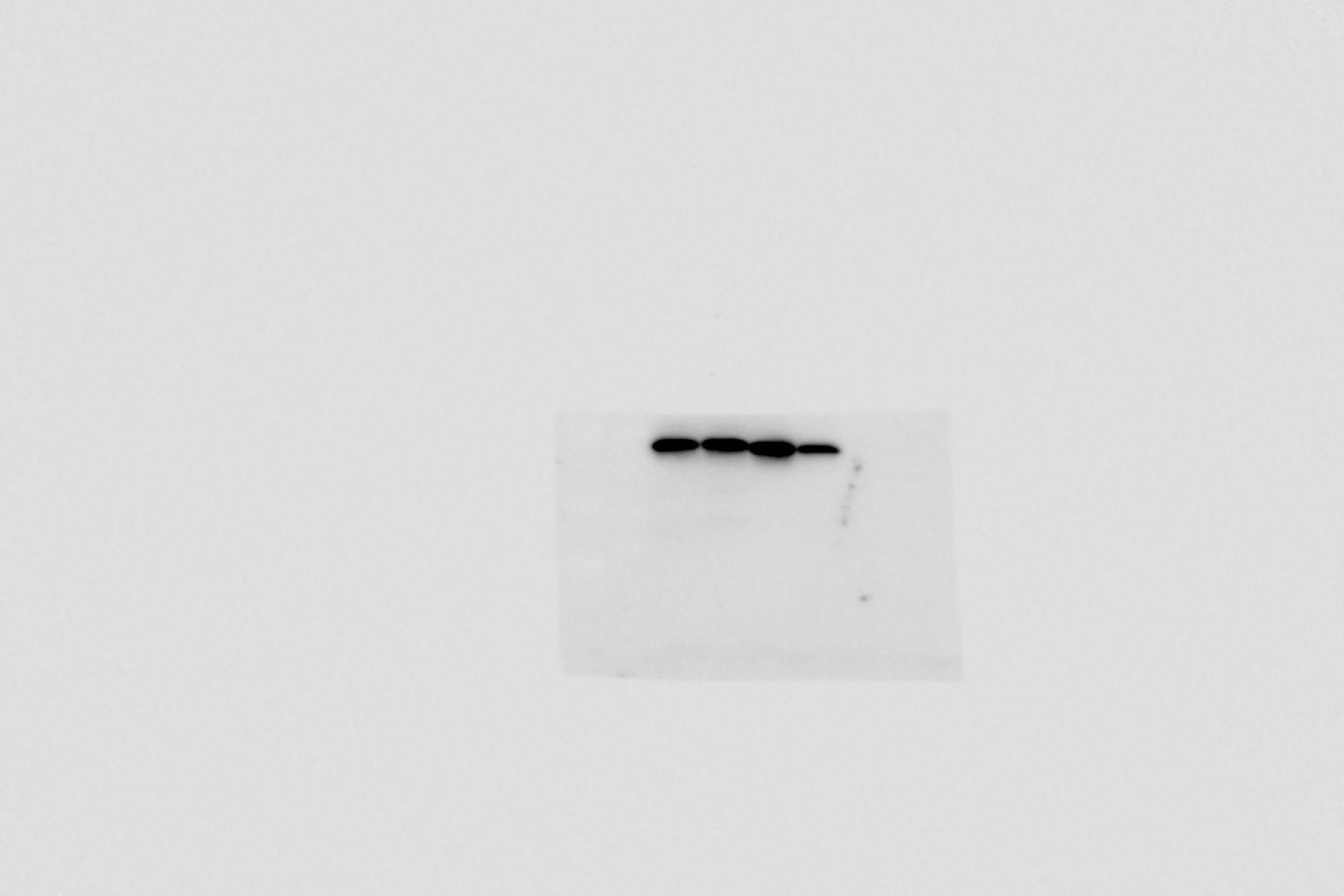
**

*Supplemental Figure S5. Experiment 3 Flagellin B, full length Western blot, uncropped*

**
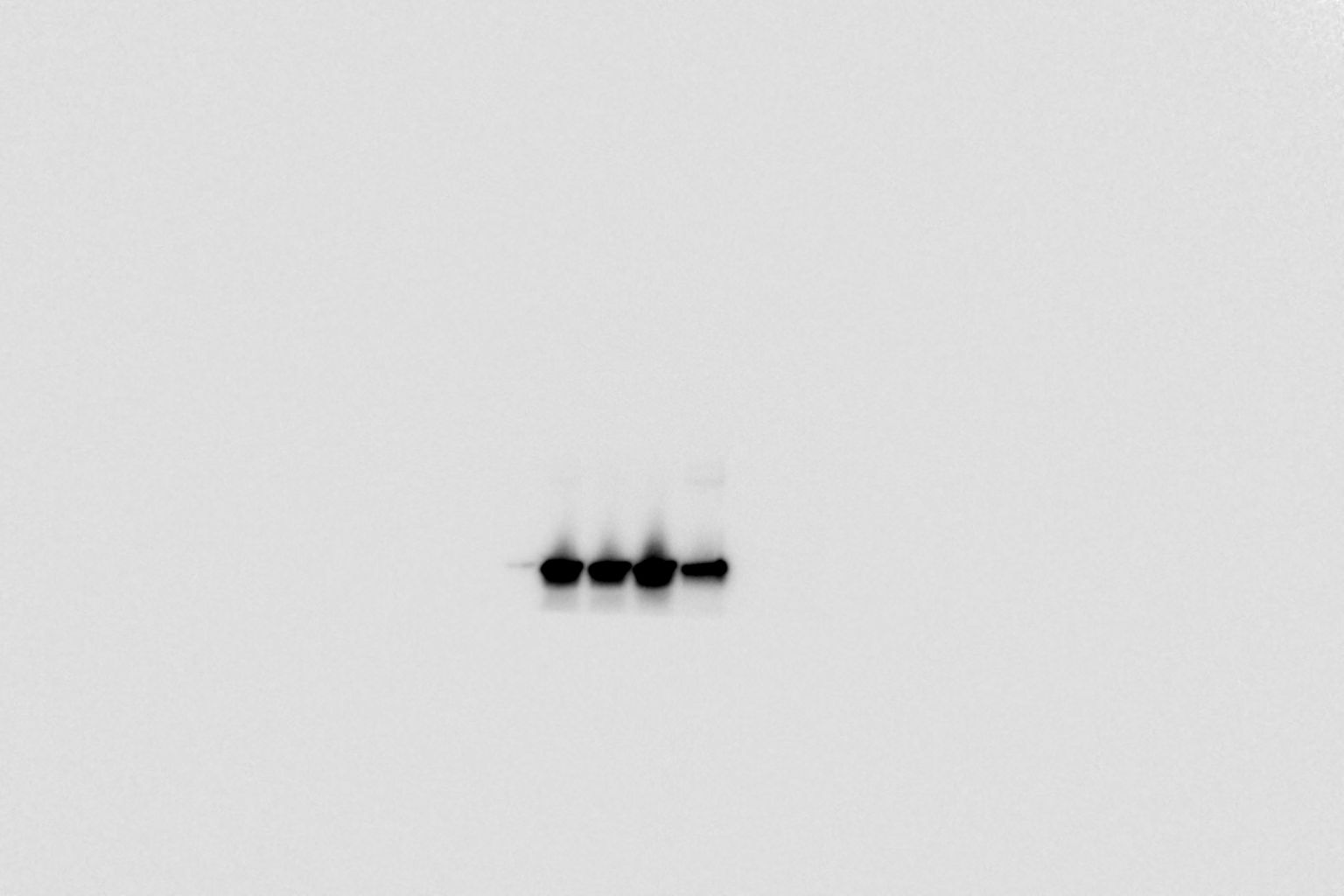
**

*Supplemental Figure S6. Experiment 3 BB0405, full length Western blot, uncropped*

**
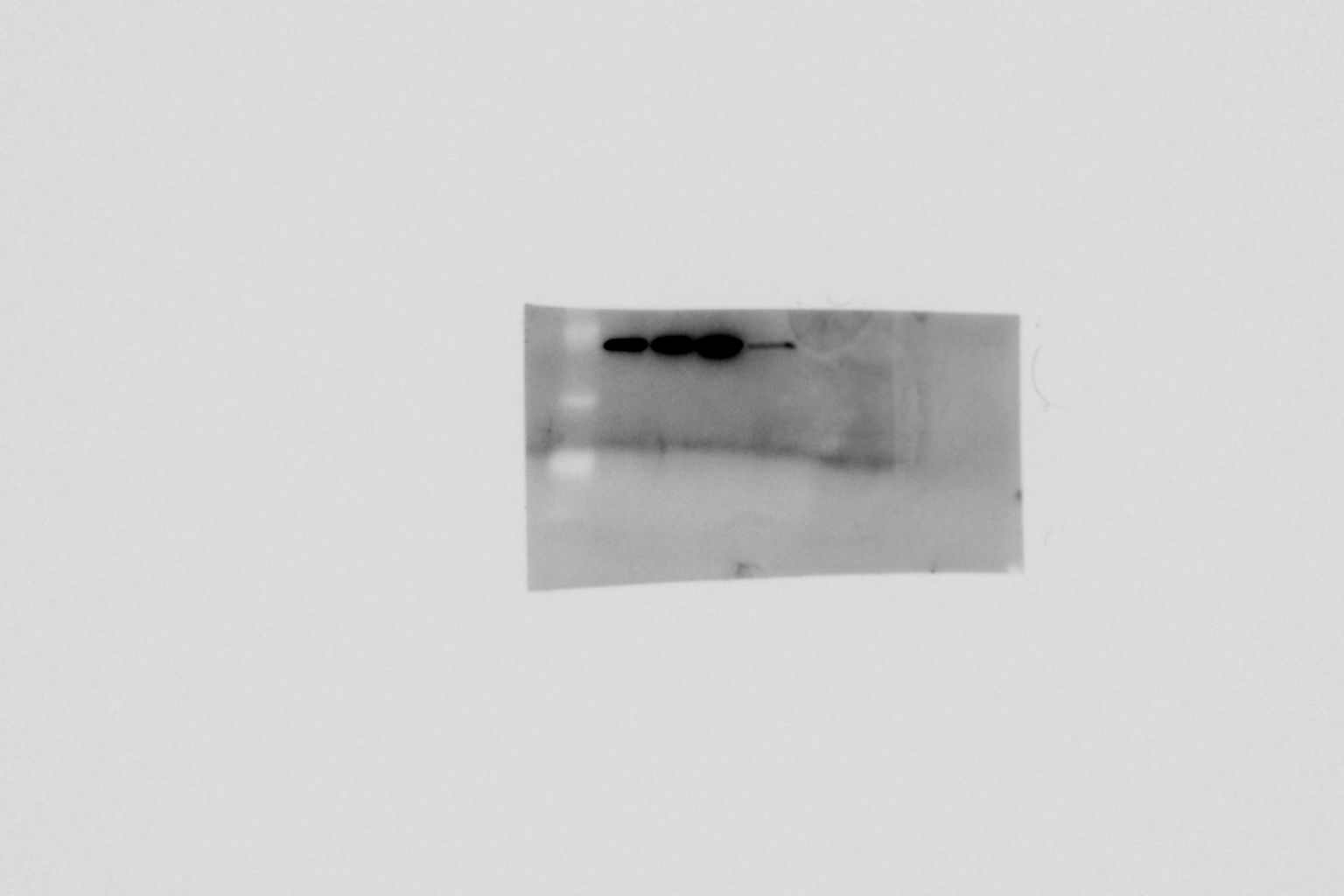
**

**Supplementary figure legends**

***Supplemental figure S1-S6. Original, full length Western blots carried out in triplicate showing expression of BB0405 in different B. burgdorferi sl strains (Borrelia burgdorferi strain B31 and Borrelia afzelii strain CB43) grown at different temperatures****.*

*Supplemental figure S1, S3, S5. Western Blots of Flagellin B, the loading control in the first, second and third replicate experiment. From left to right the bands of Flagellin B are presented of Borrelia burgdorferi B31 at 33°C and at 37°C and of Borrelia afzelii CB43 at 33°C and at 37°C (as described in Figure 3a).*

*Supplemental figure S2, S4, S6. Western Blots of BB0405, in the first, second and third replicate experiment. From left to right the bands of BB0405 are presented of Borrelia burgdorferi B31 at 33°C and at 37°C and of Borrelia afzelii CB43 at 33°C and at 37°C (as described in Figure 3a).*
